# Clinical predictors for the prognosis of myasthenia gravis

**DOI:** 10.1186/s12883-017-0857-7

**Published:** 2017-04-19

**Authors:** Lili Wang, Yun Zhang, Maolin He

**Affiliations:** Department of Neurology, Beijing Shijitan Hospital, Capital Medical University, Beijing, 100038 People’s Republic of China

**Keywords:** Myasthenia gravis, Prognosis, Ocular, Relapse

## Abstract

**Background:**

Clinical predictors for myasthenia gravis relapse and ocular myasthenia gravis secondary generalization during the first two years after disease onset remain incompletely identified. This study attempts to investigate the clinical predictors for the prognosis of Myasthenia Gravis.

**Methods:**

Eighty three patients with myasthenia gravis were concluded in this study. Baseline characteristics were analyzed as predictors.

**Results:**

Relapse of myasthenia gravis developed in 26 patients (34%). Generalization developed in 34 ocular myasthenia gravis patients (85%). Other autoimmune diseases were observed more commonly in relapsed myasthenia gravis (*P* = 0.012). Second generalization group contained more late onset patients (*P* = 0.021). Ocular myasthenia gravis patients with thymus hyperplasia progressed more rapidly than those with other thymus pathology (*P* = 0.027). Single onset symptom of ocular myasthenia gravis such as ptosis or diplopia predicted early progression than concurrence of ptosis and diplopia (*P* = 0.027). Treatment effect including glucocorticoid, pyridostigmine, thymectomy, IVIG, immunosuppressive drugs did not show significant difference between the relapsed and non-relapsed groups. The treatment outcome also showed no difference between the single OMG and second generalized groups.

**Conclusions:**

Occurrence of associated autoimmune disease can serve as a potential predictor for myasthenia gravis relapse. Either ptosis or diplopia, as well as thymic hyperplasia can predict generalization in the first six months.

## Background

Myasthenia Gravis (MG) is an autoimmune disorder targeting at neuromuscular junction by anti-acetylcholine receptor antibodies (AChR-Ab). Ocular symptoms were present in 40–50% of MG patients and ocular myasthenia gravis (OMG) developed to secondary generalized myasthenia gravis (SGMG) in 50%–80% of cases within the first 1 or 2 years [1, 2]. Studies suggested that late age of onset, high titers of anti-acetylcholine receptor (AChR) antibody and thymoma could increase the risk of secondary generalization. Early treatment with immunosuppressive drug such as corticosteroids and/or azathioprine, was suggested the possibility to reduce the risk of secondary generalization [3–6].

The clinical course of MG included remission, relapse, exacerbation and death. About 38% MG patients experienced remission [7]. One study demonstrated that early thymectomy and administration of prednisolone were more frequently seen in the complete remission cases than in the relapsed OMG patients. [7]. Anti-Kv1.4 antibody is a useful independent factor for predicting MG relapse [8]. Time of diagnosis after onset and age at onset (<40 years) were found to be predictive factors of remission. Gender seems to be an un-relevant factor for predicting MG [9]. Thymus hyperplasia was more common than other thymus pathologies in relapsed MG [9]. However, the prognostic value of thymoma on MG relapse remains inconclusive. In addition, less attention was paid on the effect of other autoimmune diseases and initial OMG symptoms of disease onset such as ptosis on one or both eyes, ptosis and diplopia concurrence or alone on both MG relapse and OMG second generalization. Our study aimed to explore the roles of these baseline clinical characters in the prediction of MG relapse and OMG secondary generalization.

## Methods

### Patients

This is a retrospective study. All patients recruited in this study were examined in the Neurology Department of Beijing Shijitan Hospital, Capital Medical University between January 2002 and October 2014. The diagnosis of MG was based on the combination of clinical and laboratory criteria [10]. Inclusion criteria consisted of fluctuating muscle weakness and one or more of the following results: (1) positive response to pyridostigmine; (2) more than 10% decreased amplitude of the compound muscle action potential in the repetitive nerve stimulation; (3) increased jitter on single-fiber electromyography (SFEMG); (4) positive AChR antibody assay. Patients with pregnancy, function failure of heart, lung, liver or kidney were excluded. The study was approved by the ethics committee of Beijing Shijitan Hospital,Capital Medical University and written informed consent was obtained from all of the patients in this study.

There were no unified criteria for the diagnosis of MG relapse [11, 12]. In this study, MG relapse was diagnosed with the reappearance of any symptoms and signs of extra-ocular, pharyngeal, neck, respiratory, axial or limb muscles weakness. The reappeared symptoms and signs should last more than 24 h. And the duration between the MG relapse and last remission should be more than 30 days. Based on these conditions, MG patients were categorized into two groups: relapsed group and non-relapsed group.

The clinical classification criteria of Myasthenia Gravis Foundation of America (MGFA) were used to rank disease severity [13]. Included MG patients were divided into MGFA Class I (OMG) and MGFA Class II-V (GMG) according to the symptoms at disease onset. OMG patients were further divided into two subgroups based on the generalization after two years of disease onset. The first subgroup remained ocular (OMG-R) and the second subgroup developed to secondary generalized MG (SGMG). SGMG patients were also divided into two subgroups based on the disease duration between the time of secondary generalization and the time of first symptom onset of OMG: ≤6 months group and 7–24 months group. Moreover, SGMG patients were also classified into three subgroups including limb group, bulbar group, both limb and bulbar group according to appearance of the first generalized symptoms.

Anti-AChR antibodies were measured by radioimmunoassay in the neuroimmunology laboratory of Peking Union Medical College Hospital. The result with the value greater than 2.966 nmol/L was considered abnormal. The diagnosis of thymic hyperplasia or thymoma was based on CT scan result. 73.5% patients undergoing thymectomy (*n* = 61) had available thymus histology result. Autoimmune thyroid diseases such as Graves’disease and antibody positive thyroid disease, were diagnosed on the basis of clinical features and laboratory examinations including serum thyroxin, serum thyroid stimulating hormone, thyroid peroxidase antibody, and thyroglobulin antibody levels. The diagnosis of Rheumatoid Arthritis was based on the guideline of The American College of Rheumatology (ACR).

### Clinical predictors

Age, gender, thymus abnormality, autoimmune diseases and symptoms of disease onset were chosen as potential clinical predictors for MG prognosis. These potential clinical predictors were compared between relapsed and non-relapsed MG groups. In addition, they were also compared between pure OMG and secondary generalized MG groups. Furthermore, we compared the drug effect including glucocorticoid, pyridostigmine, thymectomy, IVIG, immunosuppressive drugs such as azathioprine, mycophenolate mofetil, cyclosporine A, tacrolimus, methotrexate, cyclophosphamide between the relapsed and non-relapsed MG groups. The treatment effect was also compared between single OMG and secondary generalized MG groups.

### Statistical analysis

Statistical analysis was performed with SPSS 22 software (IBM, New York). Categorical variables were analyzed using the chi-square and Fisher exact test. Continuous variables were analyzed with correlation matrix. Multivariable logistic regression analysis was used to perform corrections. *P* < 0.05 was considered statistically significant.

## Results

### Predictors of MG relapse.

Eighty three patients fulfilled the inclusion criteria. There were 45 female and 38 male patients, with a median age of 39.8 ± 20.3 years old (from4 to 74 years). Fifty nine patients presented with only ocular signs and symptoms (OMG) at disease onset and twenty-four patients presented with generalized symptoms (GMG) at disease onset. These patients were divided into two groups: relapsed MG group (RMG, *n* = 26) and non-relapsed MG group (NRMG, *n* = 51) based on the prognosis after two years of disease onset. Unfortunately, information of six patients whether relapse or not was not available. The baseline clinical factors associated with MG relapse were examined by fisher exact test or Chi-square analysis. Results showed that there was no difference in gender, thymus abnormality, age, and first symptoms of disease onset between the two groups, but other autoimmune diseases such as Graves’ disease and Rheumatoid Arthritis were observed more frequently in RMG group than in NRMG group (*p* = 0.012). Treatment outcome including glucocorticoid, pyridostigmine, thymectomy, IVIG, immunosuppressive drugs did not show significant difference between the relapsed and non-relapsed groups. The common clinical features of the RMG and NRMG patients were listed in Table 1.

**Table 1:** Relapse of myasthenia gravis

	RMG	NRMG	NA	*P*-value	Time of recurrence(month)			*P*-value
					≤6	7–12	>12	
Sample size	26	51	6		11	6	9	
Age of onset(year)^b^	26	51		0.176	11	6	9	0.464
Age of onset(year)^c^								
≤ 40	10	25		0.262	4	4	2	0.219
> 40	16	26			7	2	7	
Sex								
Male	11	27		0.261	4	3	4	0.851
Female	15	24			7	3	5	
Thymus								
Normal	2	8		0.739	1	1	0	0.339
Thymic hyperplasia	12	24			6	1	5	
Thymoma	9	15			3	2	4	
NA	3	4			1	2	0	
Other autoimmune disease								
No	15	43			6	4	5	
Yes	11	8		0.012	5	2	4	0.878
Symptom of onset								
OMG	21	34	4	0.152	11	4	6	0.103
GMG	5	17	2		0	2	3	
IIa	2	0		0.307^a^	0	0	0	1.000^a^
IIb	10	2			0	1	1	
III	1	2			0	1	1	
IV	4	1			0	0	1	
Symptom of OMG onset								
Sample size	21	34			11	4	6	
Ptosis	14	21		0.443	7	4	3	0.310
Diplopia	3	3			2	0	1	
Both	4	10			2	0	2	
Ptosis								
Sample size	14	21			7	4	3	
Left	3	2		0.133	2	1	0	0.323
Right	4	14			1	1	2	
Bilateral	7	5			4	2	1	

NA: not available, Both: concurrence of ptosis and diplopia, RMG: relapse of MG, NRMG: no relapse of MG, GMG: patients presenting with generalized symptoms at disease onset, OMG: patients presenting with pure ocular MG at disease onset, Time of recurrence (month): based on the duration between the time of relapse and the time of first symptom onset of MG

^a^Difference of MGFA grades between RMG and NRMG

^b^Age at onset was not divided into two groups (statistical analysis using correlation matrix)

^c^Age at onset was divided into two groups (statistical analysis using chi-square or fisher exact test)

In addition, 26 relapsed MG patients were further divided into three groups (≤6 months group, 7–12 months group, >12 months group) based on the duration between the time of relapse and the time of first symptom onset of MG. Eleven patients relapsed within the first 6 months after disease onset. Six patients relapsed in 7–12 months after disease onset, and 9 patients were classified into >12 month group. Fisher exact test or Chi-square analysis was used to evaluate associations of baseline characteristics including gender, age of disease onset, first symptom of disease onset, thymus abnormality, and other autoimmune diseases with the relapse time of MG. We found that no statistically significant differences in above mentioned clinical features were observed among the three groups.

### Predictors of OMG generalized to GMG.

Fifty nine OMG patients were included in this study, in which the second generalization information was available in 40 OMG patients. Among the 40 patients with initial pure ocular manifestations at disease onset, 6 (15%) remained pure OMG (OMG-R) during the first two years after disease onset, while 34 (85%) progressed to secondary generalized MG (SGMG). The age of disease onset was significantly older in the SGMG group in comparison to the OMG-R group (*p* = 0.021). Gender, first symptom of disease onset, thymus abnormality and other autoimmune diseases did not show statistically significant difference between the OMG-R and SGMG groups. The treatment outcome including glucocorticoid, pyridostigmine, thymectomy, IVIG, immunosuppressive drugs also showed no difference between the pure OMG and second generalized groups. The baseline clinical features are listed in Table 2.

**Table 2:** Secondary generalization of ocular myasthenia gravis

	OMG-R	SGMG	NA	*P*-value	Time of generalization (month)			*P*-value	First symptom of generalization Limb bulbar both NA				*P*-value
					≤6	7–24	NA						
Sample size	6	34	19		21	6	7		8	13	6	7	
Age of onset(year)^c^	6	34	19	0.017	21	6	7	0.588	8	13	6	7	0.329
Age of onset(year)^d^													
≤ 40	5	10		0.021	7	1	2	0.406	2	2	3	3	0.277
> 40	1	24			14	5	5		6	11	3	4	
Sex													
Male	2	14		0.544	8	4	2	0.219	4	5	3	2	0.834
Female	4	20			13	2	5		4	8	3	5	
Thymus													
Normal	2	3		0.058	1	1		0.027^a^	2	0	0	1	0.152
Thymic	2	20			15	1			5	9	3	3	
hyperplasia													
Thymoma	0	10			5	4			1	3	3	3	
NA	2	1			0	0			0	1	0	0	
Other autoimmune disease													
No	6	21		0.077	14	3	4	0.387	6	6	4	5	0.390
Yes	0	13			7	3	3		2	7	2	2	
Symptom of OMG onset													
Sample size	6	34				34				34			
Ptosis	5	20		0.487	12	1	7	0.027^b^	4	8	2	6	0.376
Diplopia	0	3			3	0	0		2	0	1	0	
Both	1	11			6	5	0		2	5	3	1	
Ptosis													
Sample size	5	20				20			20				
Left	1	2		0.404	1	1	0	0.400	0	1	0	1	1.000
Right	1	8			6	0	2		1	4	1	2	
Bilateral	0	5			2	0	3		1	2	0	2	
NA	3	5			5				5				

NA: not available; OMG-R: patients presenting with pure ocular MG and remaining with only ocular manifestations during the first two years after disease onset; SGMG: patients presenting with pure ocular MG at disease onset and developed secondary generalized symptoms and signs during the first two years after onset; Both: concurrence of ptosis and diplopia as the first onset symptom of OMG generalization

^a^: compared between thymic hyperplasia and other thymus histology (including normal thymus and thymoma)

^b^: compared between concurrence and not concurrence of ptosis and diplopia

^c^ Age at onset was not divided into two groups (statistical analysis using correlation matrix)

^d^ Age at onset was divided into two groups (statistical analysis using chi-square or Fisher exact test)

Among the 34 patients with SGMG, 21 patients generalized within the first 6 months after symptom onset, 6 generalized within 7–24 months, whereas the generalization information was not available for the remaining 7 patients. Thymic hyperplasia was observed more frequently in the 6 months group than in the 7–24 months group (71.4% vs. 16.7%, *p* = 0.027, fisher exact test).

As the first onset symptom, either ptosis or diplopia was seen more frequently in 6 months group in comparison to 7–24 months group (71.4% vs. 16.7%), while concurrent ptosis and diplopia occurred more frequently in the 7–24 months group than in 6 months group (83.3% vs. 28.6%). First symptom of disease onset showed statistically significant differences between 6 months group and 7–24 months subgroup (*p* = 0.027, fisher exact test). Thymic abnormality and first symptom of OMG onset were useful features for predicting the development time of secondary generalization. However, there was no significant difference in gender, age of symptom occurrence and other autoimmune diseases between the two subgroups.

Furthermore, except 7 SGMG patients whose first symptom of generalization information was not available, other 27 SGMG patients were divided into three subgroups according to the appearance of first symptom of generalization. These three groups were limb group, bulbar group and concurrence of bulbar and limb group. The baseline characteristics of the patients in these three subgroups did not show statistical difference.

## Discussion

The purpose of this study is to identify risk factors that would be able to predict MG relapse and OMG second generalization. The analysis showed that only 33.8% of MG patients relapsed and 85.0% of patients with OMG onset progressed to SGMG. Concomitant autoimmune disease is observed more commonly in RMG patients (73.3%) than in NRMG patients (18.6%). The presence of concomitant autoimmune disease was identified to be a significant risk factor for MG relapse. Late age of OMG patients (>40 years) at disease onset can predict the development of SGMG. Thymus hyperplasia and initial symptom ptosis or diplopia could serve as indicators for the generalization in the first six months. Concurrence of ptosis and diplopia was a useful predictor for generalization in 7-24 months after OMG onset.

Previously many studies have addressed the predictor of relapse in MG patients. Shigeaki Suzuki et al. reported that MG patients with anti-Kv1.4 antibodies experienced frequent MG relapses in comparison to those without anti-Kv1.4 antibodies [8]. Nobuo Wakata et al. found that thymus hyperplasia occurred more commonly in relapsed cases, and early thymectomy or administration of prednisolone can lead to a reduced relapse rate in MG patients [7]. However, the limitations of these studies were that only GMG patients were included and only reappearance of ocular symptoms was considered as the relapsed symptoms. In addition, these previous studies ignored the role of autoimmune diseases in MG relapse. Our result demonstrated strong evidence that other autoimmune diseases can be used to accurately predict the occurrence of MG relapse.

In the present study, 85% of OMG patients developed second generalization. The generalization rate was higher than that in the cases of other studies reported previously [1], [2], [14]. This discrepancy may be related to the late thymectomy or other immunosuppression treatment in our study. Late age of disease onset was found to be the only factor that can accurately predict OMG second generalization. Several studies reported that immunotherapy reduced the risk of generalization and AChR antibody, thymoma were predictive of the development of secondary generalization [1, 2, 14]. Our study also demonstrated that thymoma and autoimmune diseases occurred more frequently in SGMG patients than in OMG-R patients. However, the difference was not statistically significant. This may be due to the limited number of our MG patients. In addition, our results also showed that different clinical phenotype and thymus hyperplasia predicated the time when OMG developed second generalization. To the best of our knowledge, it is the first time that the relationship of ptosis and diplopia with the time of OMG generalization was investigated.

The mechanism underlying MG relapse and second generalization of OMG remain unclear. It may be related to the modification of immune system by β2 adrenergic receptor (β2-AR) [15, 16]. β2-AR is a G protein coupled receptor with seven transmembrane domains. Our previous study implied that homozygosity for Arg16 of β2-AR gene could confer susceptibility to MG [17]. In addition, Arg16Gly was found to be more common in MG patients who also suffered other autoimmune diseases than in those without concomitant of other autoimmune diseases [18]. Results of our current study suggested that concomitant autoimmune disease was a significant risk factor for MG relapse [19]. Therefore, β2-AR gene polymorphism could contribute to MG relapse.

Moreover, β2-AR also plays a role in the prognosis of OMG. Our previous study showed that homozygosity for Arg16 mainly connected with the pathogenesis of late onset MG.[17] We also found that different genotypes at position 27 of β2-AR were related with the different thymus pathology of MG [20]. Our results suggested that late age of disease onset and thymus hyperplasia could predict the secondary generalization and the generalization time of OMG. Therefore, we speculated that β2-AR gene polymorphism might lead to the second generalization of OMG. Further studies should be conducted to address these issues in the future.

It should be pointed out that this study was a retrospective analysis done in one hospital and only examined the clinical factors that could cause MG relapse and OMG second generalization. Although treatment effect such as glucocorticoid, immunosuppressive drugs, pyridostigmine, thymectomy and IVIG between the groups showed no significant difference, future studies need further focus on the treatment including thymectomy as well as immunosuppression therapies and laboratory results such as different serum antibodies on the prognosis of MG for elucidating the pathogenesis of MG prognosis.

## Conclusions

In summary, our study demonstrated that other autoimmune diseases were risk factors that could contribute to MG relapse. Age of disease onset is a significant predictor of OMG generalization. Thymus hyperplasia and ptosis or diplopia could predict early generalization of OMG.

## Abbreviations

MG: Myasthenia gravis;
AChR-Ab: Anti-acetylcholine receptor antibody;
OMG: ocular myasthenia gravis;
SGMG: secondary generalized myasthenia gravis;
AChR: anti-acetylcholine receptor;
SFEMG: single-fiber electromyography;
MGFA: Myasthenia Gravis Foundation of America;
ACR: American College of Rheumatology
